# Ethical issues in biomedical research using electronic health records: a systematic review

**DOI:** 10.1007/s11019-021-10031-6

**Published:** 2021-06-19

**Authors:** Jan Piasecki, Ewa Walkiewicz-Żarek, Justyna Figas-Skrzypulec, Anna Kordecka, Vilius Dranseika

**Affiliations:** 1 Department of Philosophy and Bioethics, Faculty of Health Sciences, Medical College, Jagiellonian University, Michalowskiego 12, 31-126 Krakow, Poland; 2 HTA Registry Sp. z o.o. Sp. K, Herzoga 15, 30-252 Krakow, Poland; 3 Fundacja Optimum Pareto (Optimum Pareto Foundation), ul. Celna 6/9, 30-507 Krakow, Poland; 4 Institute of Philosophy, Vilnius University, 9/1 Universiteto, 01513 Vilnius, Lithuania

**Keywords:** Electronic health records, Learning healthcare system, Embedded research, Ethics, consent, Systematic review

## Abstract

Digitization of a health record changes its accessibility. An electronic health record (EHR) can be accessed by multiple authorized users. Health information from EHRs contributes to learning healthcare systems’ development. The objective of this systematic review is to answer a question: What are ethical issues concerning research using EHRs in the literature? We searched Medline Ovid, Embase and Scopus for publications concerning ethical issues of research use of EHRs. We employed the constant comparative method to retrieve common ethical themes. We descriptively summarized empirical studies. The study reveals the breadth, depth, and complexity of ethical problems associated with research use of EHRs. The central ethical question that emerges from the review is how to manage access to EHRs. Managing accessibility consists of interconnected and overlapping issues: streamlining research access to EHRs, minimizing risk, engaging and educating patients, as well as ensuring trustworthy governance of EHR data. Most of the ethical problems concerning EHR-based research arise from rapid cultural change. The framing of concepts of privacy, as well as individual and public dimensions of beneficence, are changing. We are currently living in the middle of this transition period. Human emotions and mental habits, as well as laws, are lagging behind technological developments. In the medical tradition, individual patient’s health has always been in the center. Transformation of healthcare care, its digitalization, seems to have some impacts on our perspective of health care ethics, research ethics and public health ethics.

**Supplementary Information:**

The online version contains supplementary material available at 10.1007/s11019-021-10031-6.

## Introduction

An electronic health record (EHR) is a technological innovation that consists in digitization of an individual patient’s health information. EHRs have already changed the landscape of biomedical research (Häyrinen et al. 2008; Foley and Fairmichael 2015; Evans 2016). The digitization of a paper-based health record alters its *accessibility*. A paper health record can be accessed only physically in the hospital archives, whereas an EHR can be accessed electronically by multiple authorized users from remote locations (Häyrinen et al. 2008; Evans 2016). Easy, speedy, and relatively cheap access to health information is the main fuel of any learning healthcare system (LHS) (Evans 2016). In an LHS, a process of generating scientific knowledge is embedded in practice: health information produced in the course of providing and receiving healthcare, is collected and analyzed; and then the subsequently generated knowledge is applied to current practice; the cycle starts again (Friedman and Macy 2014). However, secondary use of an EHR beyond the scope of clinical care raises a series of ethical questions.

These ethical questions concern the necessity of requirement of informed consent (Helgesson and Eriksson 2008; Hansson 2010), and the limits of physician–patient confidentiality in the context of embedded research and risk of being re-identified (Sweeney 2000; El Emam et al. 2011, 2015; Simon et al. 2019). Data extracted from an individual EHR, if leaked, can be potentially used to deny health services, insurance, and bank products, as well as to stigmatize individuals and groups. However, our review shows also that embedded research poses ethical challenges for healthcare professionals and healthcare institutions who are not always comfortable with sharing health data for the purpose of research, deeming that it could undermine relationships with their patients and their reputation (Simon et al. 2017). Moreover, EHRs can be also considered as an instrument of patients’ empowerment and instrument of patients' contribution to progress in medicine and protection of public health.

Laws and guidelines regulating the collection and processing of personal and health information can differ from country to country, but most developed economies have extensive regulations concerning data. However, ethical and practical problems seem persistent despite the existing numerous laws. In the US, the Ascension and Google’s Project Nightingale sparked public outrage (Pilkington 2019). In the UK, the care.data project faced vigorous public resistance (Anderson 2015; Hall 2016), and reportedly a similar project in Denmark also was an issue of controversy (Skovgaard et al. 2019). In all these cases, research activities, even though they were, strictly speaking, legal, were rejected by the public and became politically infeasible. These examples demonstrate that following the laws is not always sufficient for ethical action.

Moreover, legislation does not fully keep pace with technological development and private sector activities (Aicardi et al. 2016; Rumbold and Pierscionek 2017; Chassang 2017; Cohen and Mello 2018). In addition, individuals are not aware of existing safeguards (Hill et al. 2013). Therefore, it seems there is a need for ethical clarity and consensus among policy makers, healthcare providers, software developers, researchers, and patients in regard to ethical standards for research use of EHRs. Building an ethical and conceptual framework for trustworthy LHSs powered by EHRs is still ahead (Evans 2016).

The regulatory effort should be preceded by an impartial, maximally transparent and comprehensive process of evidence gathering. A systematic review of literature helps to meet these standards, providing decision-makers with a spectrum of ethical challenges that are currently discussed and should be taken into consideration (Klingler et al. 2017).

The ethical issues of EHRs in the context of biomedical research have not as yet been the subject of systematic literature review. However, the ethical problems concerning related questions, such as research in digital databases (Aitken et al. 2016), public health surveillance (Klingler et al. 2017), LHS (McLennan et al. 2018), ownership of health data (Mirchev et al. 2020), and public attitudes towards EHRs (Hill et al. 2013, Skovgaard et al. 2019), have been recently reviewed in a systematic manner. Our review is intended to fill this gap and answer the question: What ethical issues concerning EHRs in the context of biomedical research are discussed in the literature? We hope that our results can be useful for professionals working under various legal regimes, concerning research involving humans, privacy protection and data processing. The results of this literature review can be a point of departure in a search for practical policy solutions. Regulators, software developers, electronic security specialists and researchers who are involved in designing policies and laws for healthcare systems may use it to determine if their policies cover all ethical aspects of the EHRs present in the literature. Moreover, this review can also be informative for institutional and individual healthcare providers who struggle with policies, procedures and day-to-day decisions concerning access and sharing of patients’ EHRs by giving them an exhaustive summary of ethical issues that should be addressed. We hope this review of literature can be a starting point for further normative analyses and research, especially into problems that have not been sufficiently discussed in the literature but can be important for the development of a trustworthy LHS.

## Methods

The protocol of the review was registered on Prospero in advance (CRD42018094526) and we followed the PRISMA protocol as far as it is applicable to a qualitative review: we did not follow the recommendations for data synthesis, and we did not conduct meta-bias assessment.

### Eligibility criteria

In our analysis, we included all papers that met the conjunction of three criteria: papers that (i) discuss ethical issues concerning (ii) the use of EHRs (iii) in the context of either biomedical research, or learning healthcare systems, or quality improvement activities. We defined the term *ethical issue* as roughly referring to one of the four ethical principles of biomedical ethics distinguished by T. L. Beauchamp and J. Childress: the principle of respect for autonomy, the principles of beneficence, non-maleficence and justice (Beauchamp and Childress 2013). We noticed that every general principle also covers more specific ethical issues. For instance, the principle of respect for autonomy covers the principle of respect for privacy, the requirement of informed consent, and the obligation to restrict disclosure of health information. Therefore, we included a paper if it discussed either general or more specific ethical issues that can be subsumed under one of the four principles. However, the four principles and their derivatives were considered only as a signal for ethical issues. As we explain later, we did not limit our analysis only to those principles.

We understood the term *electronic health record* (EHR) as digitized health information of an individual patient which is stored electronically in a healthcare system: a single medical facility, a chain of facilities, or a national healthcare system. The terms *biomedical research, learning healthcare* and *quality improvement* are construed as activities generating generalizable knowledge in the context of healthcare. We accepted peer reviewed articles, book chapters, reports, guidelines, commentaries, and letters to the editor published in English. We excluded all papers without sufficient amount of ethical deliberation, as well as conference abstracts and newspaper articles. The term “sufficient amount of ethical deliberation” was understood as an amount of meaning that can be captured in a separate subcategory in a process of constant comparative reading. To ensure maximum objectivity of this element, two coders were involved in identifying whether inclusion criteria as described above apply to a given publication.

### Sources and search strategy

We conducted systematic searches in Medline Ovid, Embase, and Scopus databases, with no time limitation on 22/03/2018, using the subscription available at  Jagiellonian University Medical College. The search strategy for each database is presented in supplementary materials (Supplement 2. *Search string*) and in the published protocol (Prospero CRD42018094526).

### Data management

Search results were exported to an Endnote database for automated duplicate screening. EWŻ manually excluded all duplicates that were not removed during the automated screening. All records were then subsequently exported to a Microsoft Word document and a screening protocol was created. EWŻ made the Word document and the protocol available through a web-cloud to all authors involved in screening procedures. The title and abstract screening were preceded by the preliminary training phase. The aim of the training phase was ensuring that all authors and contributors understand the eligibility criteria in a uniform manner. In this phase, contributors PB and ES (under EWŻ’s supervision) and JP screened the first 100 records for the eligibility criteria in order to verify the consistency of the approach used.

### Selection process, data collection and data analysis

In the first phase of our review, the titles and abstracts were screened for eligibility criteria by two referees (contributors PB and ES under EWŻ and AK’s supervision). All disagreements were resolved by JP. Previously undetected duplicates were excluded as well. Papers meeting the eligibility criteria were then downloaded and underwent full-text screening by JP and EWŻ. Any disagreement was resolved by discussion and consensus. The eligible papers were subsequently analyzed by JP, EWŻ, and JFS.

### Qualitative methodology

We conducted qualitative analysis using the constant comparative method (CCM) that has an inductive character and consists of reading a text with an intention to capture the main recurring units of meaning (Boeije 2002; Dye et al. 2000; Gibbs 2009). When subsequent materials within the sample are analyzed, the units of meaning can be generalized and remodeled. The outcome of the entire analysis is a list of categories discerned in the papers (see: Table 1, and Supplement 1. *The Full Grid*). The qualitative analysis was conducted by two pairs of coders to enhance the objectivity of the process. JP created a draft grid of categories and then subsequently discussed it with EWŻ, JFS and VD. After agreeing to the final version of the grid, the authors (JP, EWŻ, and JSF) independently coded the papers.

**Table 1:** Grid of categories

Sl	Category	N
A. Rationale for research using EHRs		
1	Public interest	43
2	Value of EHR research	36
3	Justice of the healthcare system	8
4	Private sector profits	7
B. Factors affecting research use of EHRs		
5	Obstructive regulations	31
6	Regulatory facilitations and institutional support	27
7	Technical difficulties with EHR database implementation	25
8	Factors hindering informing participants about research and obtaining consent	22
9	Public awareness, experience, opinions and attitudes	24
10	Researchers' attitude	3
C. Data management		
11	Safety and security	36
12	Levels of data identifiability and research	41
13	Special types of data and their protection	13
14	Data quality, quantity, and integrity	26
15	Data ownership, management, and curation	15
16	Meaningful data sharing	19
17	Data storage, extraction, and data transfer	30
18	Legitimacy of uses and users of EHR data	22
D. Impact of digitalization on healthcare system: providers' operations and patient engagement		
19	EHR-based research and changed professional relationships within health care institutions and between various institutions	19
20	EHR-based research and the practice-patient relationship	11
21	Medical staff and ethical responsibility in the context of EHR and EHR-based research	17
22	Additional workload for staff and patients	12
23	Pivotal role of patients’ contribution	18
24	Ideas for the future: digital patient-citizenship	14
E. Risks, harms and burdens of research with EHRs		
25	Risk to privacy	39
26	Compromised patients' autonomy	21
27	Dignitary harm—being wronged	9
28	Harmful or wrongful use of data	11
29	Legal harm	2
30	Psychological harm	3
31	Information risk	13
32	Risk of exploitation	12
33	Undue pressure to participate	2
34	Undermined trust	14
35	Compromised care	5
36	Group mediated risks	15
37	Financial conflict of interest	9
38	EHRs as a challenge and potentially negative influence on healthcare practices, staff, and patient-provider communication	13
39	Risks to healthcare provider	3
F. Measures for subject protection		
40	Independent review	26
41	Informed consent or authorization of data use	43
42	Legal, ethical and professional regulations	19
43	Risk assessment and risk minimization, non-maleficence	14
44	Primary care provider consent	2
45	Community/panel of patients consent	2
46	Provider consent mechanism	2
G. Types of consent		
47	Initiation of contact	4
48	Options on the continuum from no consent via opt-out, to explicit and informed consent	27
49	Verbal consent, written consent	14
50	Broad—narrow consent	8
51	Interactive consent and granularity	16
52	Retrospective consent	6
53	Proxy consent	4
54	Assent	2
H. Content of consent		
55	Data management: purpose, future use, storage and data sharing	15
56	Security measures	12
57	Benefits, risk and burdens	10
58	Commercial application of data	9
59	Communication of results	4
60	Sources of research funding	2
I. Reasons and motives for participation in EHRs-based research		
61	Benefits of making one's EHR available	7
62	Moral values and obligations	13
63	Trust in people and institutions inviting one to participate in EHR-based research	13
64	Personal cirumstances and characteristics	14
J. Emotions experienced as a result of reflection on EHRs and/or participation in EHR-based research		
65	Emotions indicating respondents' positive attitude towards EHR-based research	9
66	Emotions indicating respondents' negative attitude towards or doubts about EHR-based research	12
67	Emotional spectrum and ambivalence	5
K. Ethical values, rights and obligations		
68	Autonomy, control over personal data, dignity, confidentiality and privacy	49
69	Information, public education and engagement	22
70	Principle of beneficence	17
71	Principle of justice	12
72	Research integrity	5
73	Optimal health care and clinical judgment	3
74	Right to compensation	1

For the full Grid with the descriptions of the categories and (N) their occurrence in the articles see Supplement 1. *The Full Grid*

An extraction chart of quantitative data from empirical studies was prepared by JP and consulted with JFS. Then quantitative data were independently extracted by JFS and JP in accordance with the chart. The data was then summarized in a narrative form and presented in Table 2. Narrative summaries of qualitative research were created by JP and consulted with JFS (Table 2).

**Table 2:** Narrative summaries of empirical studies

First author, year	Journal	Country	Number of subjects, subjects characteristics	Research design	Main findings
Botkin et al. (2014)	Journal of Community Genetics	USA	131, > 18, General population,	Qualitative study, focus groups—semi-structured discussion following the video on EHRs research	Initially the majority of participants did not know that EHRs were used for the purpose of research. When participants of the study were presented with educational materials by researchers, they expressed strong support towards research. Also the majority of participants preferred opt-out form of consent to research, referring to public benefits as justification. Nevertheless, some participants wanted to give informed consent before their EHRs are used in research. The participants stress the need for proper public education about the EHRs research
Clerkin et al. (2012)	Family Practice—The International Journal for Research in Primary Care	Ireland	35, > 18, patient in primary care setting,	Qualitative study, focus groups	Patients supported the idea of their records being used in research for the greater good. However patients are concerned about possible risk to their privacy and harmful effects, such as loss of job or insurance (mainly men) or social discomfort and embarrassment (women). Patients want to control their records, and they are willing to give their broad consent to a specific, chosen type of research; then they do not have to be informed on a study-by-study basis (broad consent)
Geissbuhler (2013)	International Journal of Medical Informatics	21 European countries	> 100 Participants of 2012 European Summit on Trustworthy Reuse of Health Data: decision makers, scholars, representatives of patients, industry, and European Commission	Participatory research: discussing scenarios in breakout sessions	Benefits of reuse of data outweigh possible risks. The most important challenge is to build a trustworthy framework for reuse of electronic health records. In such a framework involvement of citizens plays the essential role and they deserve to be “fully informed”. Governments should regulate and oversee reuse of patients’ data. Lack of a unified framework for reuse of electronic data in the EU entails increasing costs of research, missing economic opportunities for European countries (pharmaceuticals, health technology and devices, eHealth solutions), and undermines patients’ safety
Grande et al. (2013)	JAMA Internal Medicine	USA	3064, > 18, representative online panel	Quantitative study, Internet survey, experimental design using scenarios, likert-scale, check-box	Among the factors influencing participants’ willingness to share health information the purpose of use is more significant (63.4% importance weigh) than the type of data user (university hospital/drug company/public health department; 32,6% importance weigh) and the level of sensitivity of data (low sensitivity “medical history”, high sensitivity: “personal genetic test predicting risk of cancer”; 3.1% importance weigh). People favor research use of data over healthcare quality studies and marketing. People trust university hospitals more than the government, drug and insurance companies. Social and racial factors are associated with trust: non-Whites are more willing to share their health information for non-research purposes and those who do not have secure access to health care are less supporting of secondary use of data
Grande et al. (2014)	Annals of Internal Medicine	USA	3064, > 18, representative online panel	Quantitative study, internet survey, experimental design using scenarios, likert-scale, check-box	Respondents agree that the use of personal information for the sake of research is appropriate, but they have ambivalent feelings about research using personal data without consent. Participants rate the acceptability of use of EHRs for the purpose of research much higher than for the purpose of marketing. Results of the study indicate that the purpose of research using EHRs is more important than informed consent, because participants see unconsented research use as more appropriate than consented marketing use (5.65 vs. 4.52; difference = 1.13; 95% CI 0.87, 1.39, using the Likert scale: 1 = not at all appropriate, 10 = very appropriate)
Jones et al. (2017)	Journal of Oncology Practice	USA	32, > 18, cancer patients	Qualitative study, semi-structured in-depth interviews	Cancer patients felt uncomfortable with a permissive system that offers fewer notifications about when and how their data are used (care, research, marketing), by what kind of user (physician, researcher, insurance company), and if sensitive data are included. They preferred opt-in form of consent, as well as they favored stringent oversight especially over the use of data by insurance and pharmaceutical industries. They were more comfortable (more trustful) with the use of their data by physicians and researchers
Kim et al. (2017)	BMC Medical Ethics	USA	800, > 18 not fully representative sample	Quantitative survey, phone interview, questionnaire, likert-scale	The majority of respondents declare willingness to consent to sharing their medical information for the sake of research (74,8%), and a slight majority values more research benefits than privacy (50.6–46.8%). However, the majority of participants value control over their EHRs more than research benefits (69.8–26.9%) but at the same time they want to have control over their medical records. Participants' willingness to share their date for the sake of research is linked with education (higher level of education is positively correlated with higher likelihood of consent), race (Hispanic, black, Asian and others were less likely to consent than Whites), familiarity with EHR (those who already have EHR are more likely to consent) and belief that EHR improve quality of healthcare (those who believe in a link between EHR research use and quality of healthcare are more willing). Health status, as well as previous experience with technology are not predictors of consent
King et al. (2012)	International Journal of Medical Informatics	Australia	700, > 18, representative group	Qualitative survey, phone interview, questionnaire, likert-scale	Most respondents declare that research is important and are willing to share their health information. The majority of participants believe that their consent should be sought for use of their health information for any purpose other than medical treatment (92%). Majority of respondents declare concerns over privacy (66%) and only a slight minority of those who are concerned feel reassured when extra security measures are put in place (16%). Information that EHRs are de-identified reduces concern about consent. However, many respondents are not aware of a risk of re-identification. Younger, 18–19 y (51.4%) and older, 60 + years old (53.4%) respondents less frequently declare being concerned about privacy than those in between age groups 20–59 years old (73.3%)
			23, > 18, four groups: elderly patients, frequent receivers of health care; persons with higher education; persons “with different ethnic background”, a mixed group, > 18	Qualitative study, focus groups	Participants expressed a wide range of attitudes towards EHR's secondary use: from approval and acceptance to privacy concerns. Majority of participants thought that medical research is important, although some were afraid that it may compromise their privacy. Also their view on consent requirement was diverse: some claimed that consent is not necessary, others required consent and very detailed information. Participants expected respect, they wanted to control their data, and to have the ability to track the users, in case something goes wrong
Nair et al. (2004)	Journal of Health Services Research & Policy	Canada	17, > 18 patients in primary care setting,	Qualitative study, semi-structured interviews	Patients generally did not know that their records were already used in research. Majority of patients (13) wanted to be asked for consent and be informed about the details of the study. Patients thought that control over their records and proper communication between them and their healthcare provider is crucial for being treated respectfully, not merely as an object of research. Patients recognized both the value of research and the need for balancing their consent preferences with time pressures in the clinical encounter, nevertheless they were concerned about the privacy of their records. They thought that health data should not be tradeable, especially to pharmaceutical and insurance companies
Riordan et al. (2015)	International Journal of Medical Informatics	UK	3157, > 18	Questionnaire: yes/no, check-box	People want to be asked for consent before their EHR is accessed for research with identifiable data purposes (91%). A slight majority wants to be asked even before research use of their anonymized data (51%). 41% of the respondents were not aware of EHRs’ existence prior to taking part in the study. Support for explicit consent is associated with ethnicity and nationality (non-White and non-British), lower computer skills, poorer knowledge about the system and lower level of education
Stevenson et al. (2012)	Family Practice—The International Journal for Research in Primary Care	UK	50, > 18 patients, in Health Research Support Service Study; 6 focus groups, and 17 interviews with patients, and 6 interviews with staff	Qualitative study, focus groups and interviews	Patients and staff generally support research using EHRs, however opt-out type of participation elicited more split responses from both participants and staff. Patients and staff appreciated the benefits of the opt-out option (inclusiveness and effectiveness of research), although they were concerned whether it gives a fair opportunity to consent for participation. Patients expressed their concerns about the safety of data and possible misuse of data by private companies. Trust in healthcare institutions was an important factor for participation
Stevenson (2015)	BMC Health Services Research	UK	68, Patients—approached in Health Research Support Service Study (50), stakeholders (11), staff (7)	Qualitative study, focus groups and interviews	This is a qualitative evaluation of a pilot Health Research Support Service (HRSS) study, designed to assess feasibility of the Clinical Practice Research Datalink—the NHS (England) observational data and interventional research service. Topics emerged from thematic analysis were subsumed under four broader categories: understanding of the study, commitment and engagement, ‘work’ involved in participation, reflections on the process. Patients and staff had problems with understanding the study, e.g. patients did not know that they were not selected and that their action was required to opt-out. Patients and staff did not know that data will not be anonymized prior to leaving the practice system. Patients overlooked or did not pay attention to the information letter about the study, staff did not pay attention during the staff meetings. Patients and staff were committed to the study not due to engagement with HRSS, but because they trust NHS or their provider. Patients and staff acknowledged the social value of research, but were concerned about the fact that identifiable data are used and opt-out option is used as a proxy for consent. Stakeholder saw the value of the project for safety, efficacy and advances in medical sciences
Weitzman et al. (2010)	Journal of Medical Internet Research	USA	151, > 18	Quantitative survey, Internet survey, questionnaire, check-box, Likert-scale	Users of Personal EHRs are not fully aware of the possibility of granular sharing of health information. Personal EHR allows a consumer to determine restrictions on sharing their record in terms of content (e.g. making available only some specific elements of a PHER) and time. Participants declared that they would be more willing to share their data if they were given the option to share it in a granular manner. Almost all respondents are willing to share their health information for the purpose of research (91%). Majority want to strictly control access to their records and want an opt-in form of consent for research (59%)
			30, > 18, patients, early users of personal EHRs,	Qualitative study, semi-structured focus groups and interviews	There was no clear preference for either opt-in or opt-out as a form of inclusion into research. Participants expressed their demand for information about and explanation of risk and benefits involved in research. Participants were willing to put some limits on information: time limits for use, or the limited scope (granularity). Guaranteeing privacy but not anonymity decreases willingness to share, however security audits increased trust and willingness to share data. Emergency research also increases willingness to share data
Weitzman et al. (2012)	BMC Medical Informatics and Decision Making	USA	261, > 18 or parents of patients, Personally controlled health record users,	Internet survey, questionnaire, check-box, open-ended question	People want to have personal EHRs and they like to have control over and access to their health information although they have limited knowledge about personal EHRs’ functioning. Respondents declare that they see value in EHRs in seeing records at any time (93.8%), monitoring health problems (68.9%), sending EHRs to other institutions (51.5%), participating in research (26.6%), participating in health/wellness activities (21.6%). They are more willing to share their information with public health authorities (63.3%) than with other providers (54.1%). The respondents are afraid of discrimination, lack of anonymity and government insensitivity. They do not want to share their sensitive (esp. financial, family and mental health status) information
Willison et al. (2008)	Journal of the American Medical Informatics Association	Canada	1230, > 18, a sample with different characteristic than a general population	Phone interview, scenarios, likert-scale	Most people support the use of EHRs and agree that beneficial research is more important than privacy. Respondents believe that “it is okay” to use EHR for the purpose of marketing campaigns (35%), to produce also profitable products (60%), improve quality of care (86%), track communicable diseases (89%). Majority want to be asked for their consent for access to health information: every time an EHR is accessed (32%), regularly renewed general permission (23%), notification (24%), and access without consent (12%). Among those who support the use of EHR, the minority (27%) think that consent is not necessary for automated extraction of data. The respondents declare trust in data institutes, hospitals, disease foundations, and university researchers, but tend not to trust insurance companies. Government and drug companies are trusted by half of respondents

### Possible biases and limitations

This study has some limitations. We did not search databases other than those listed above. For example, we did not search Google Books and Google Scholar. Although the latter is well-known for containing an abundance of grey literature, a search in this database is not fully replicable (Haddaway et al. 2015, Piasecki et al. 2018). Therefore, our choice probably decreased the sample size, but at the same time it enhanced transparency and replicability of searches, which are crucial for systematic review. Moreover, the main purpose of a qualitative study is to obtain a sufficiently rich sample and our study meets this criterion, providing us with an abundance of diverse data.

It can be argued that the four-principle ethical framework does not offer an adequate set-up for the ethical problems of EHRs in particular or that it does not offer a completely neutral approach to ethical problems in general. The former objection can be elaborated in the following way: there are new frameworks devised for the purpose of tackling ethical and legal issues of Big Data, such as the solidarity-based approach (Prainsack and Buyx 2013) and the concept of group-privacy (Taylor et al. 2016). These new approaches seem to be more useful for analyzing the specific issues of data analysis. However, in response it could be pointed out that a qualitative systematic review is better served by a set of general inclusion criteria. This increases sensitivity of a search and is more consistent with the goal of identifying possibly the broadest spectrum of ethical issues.

Replying to the second objection, it can be said that probably there is no ethical framework that is completely culturally neutral and fully universal. Nevertheless, the four-principles framework allows to map the most general ethical tensions present in biomedicine that can occur in clinical settings at every latitude; the tension between: individual interests and interest of society (respect for autonomy *versus* justice), prospect of benefit and the risk of harm (beneficence *versus* non-maleficence), individual will and medically defined wellbeing (autonomy *versus* beneficence). Therefore, this is a useful instrument for detecting the ethical content in a paper, and as mentioned above, we consider the four-principle framework only as a signal for ethical issues. Using the Beauchamp-Childress framework does not imply adopting any specific ethical position. Moreover, our analysis has an inductive character. Therefore, we consider as ethical issues also those issues that were considered ethical by the authors of the analyzed papers, and in our results we go beyond this framework. Furthermore, the Beauchamp-Childress framework has already been successfully used in other systematic qualitative reviews (Klingler et al. 2017; McLennan et al. 2018; Strech et al. 2013).

Another limitation of our study is that we did not include papers that were not published in English and, therefore, we could have lost some important ethical aspects of EHRs that appear in other cultures, outside the Anglophone world. We agree that the full picture of ethical issues concerning EHRs must be supplemented by cross-cultural studies. However, it should be noted that in the case of non-English publications, there may be uncertainty in the correct interpretation of ethical aspects, precisely because of language or cultural differences.

## Results

### Search results

**Fig. 1:**
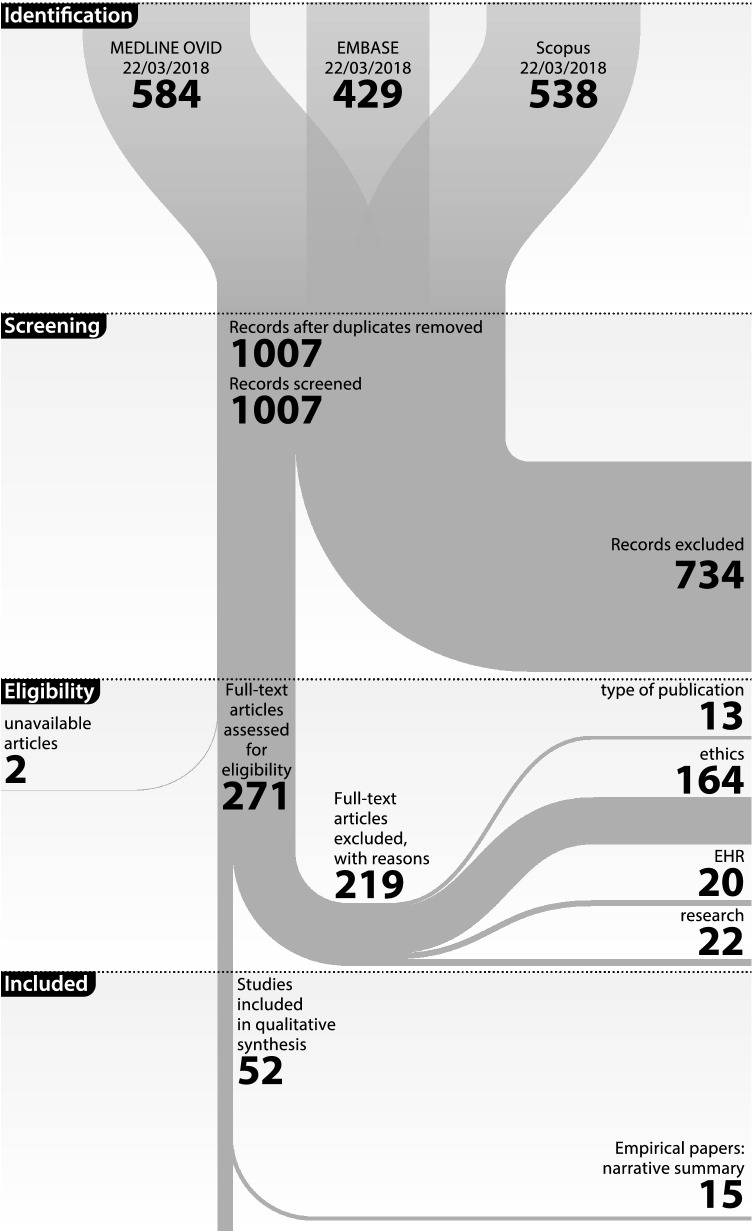
PRISMA flow diagram

Searches in Medline Ovid, Embase, and Scopus (22/03/2018) identified, after duplicates had been removed, resulted in 1007 potentially eligible documents (see Fig. 1 presenting the results of all phases). The title-abstract screening resulted in 271 documents for full-text screening (2 were unavailable). The final sample of papers meeting the inclusion criteria was 52 documents. They were divided into 3 groups based on the nature of a paper. The first group consists of 37 policy papers that either discuss a certain policy proposal and provide ethical justification for it or discuss general ethical framework for EHRs research and suggest certain policy proposals, or describe how to meet policy and ethics requirements in conducting research using EHRs. The term “policy paper” refers exclusively to the content of a text; a policy paper could be a commentary, an original study, or a book chapter. The remaining 15 empirical papers are a sample of 9 papers containing qualitative data analysis and a sample of 8 quantitative data summaries, which means that 2 papers presented mixed qualitative and quantitative research. We applied the CCM to devise a grid of categories (Table 1) based on all 52 papers. In addition, we provide a narrative summary of 15 empirical papers (Table 2).

### Qualitative analysis results

In the qualitative synthesis, we distinguished 74 specific categories that cluster around 11 general topics (main categories are marked by capital letters A–K, specific categories are marked by a capital letter and a number: A1-K74, see Table 1, and Supplement 1. *The Full Grid*—this last document allows to estimate the frequency of each category in all the papers and saturation of individual papers with ethical issues. One interested in a particular issue can also locate relevant papers via list of references in the Supplement 1). Below we describe both main categories and subcategories.

#### Rationale for research using EHRs (A1–A4)

The first distinguished category is related to the intrinsic and instrumental values of EHR-based research that are mentioned in the included papers. The first reason for implementing EHR-based research is public interest (A1). EHR-based research is deemed instrumental in improvement of public health as well as in increasing efficacy and efficiency of healthcare. The second reason is efficiency and efficacy of EHRs research (A2): EHR-based research is relatively inexpensive and this kind of research can circumvent shortcomings of randomized clinical trials by providing researchers with data about all groups of patients and in that way contributing to generalizable knowledge. The included papers also discuss the fact that research with EHRs can give us a comprehensive picture of a healthcare system, and therefore allow for a more equitable allocation of resources (A3). Finally, the included papers discussed the fact that EHR-based research also promises benefits to the private sector: private hospitals, as well as insurance and technological companies (A4).

#### Factors affecting research use of EHRs (B5–B10)

Even though EHR-based research is valuable, its implementation is not always easy. The papers we analyzed discussed various factors that can either facilitate this kind of research or obstruct it. The most frequent factor that impedes EHR-based research is regulations that put a burden of administrative work on researchers (B5). This could be especially cumbersome in multicenter international research, where there are unharmonized regulations and multiple research ethics committees. Moreover, a research project can fall within the blurred lines of the distinction between research and quality improvement, making it even more difficult for researchers and research ethics committees to decide which regulatory framework should be used.

Policy makers, however, are well aware that regulations can put too heavy a burden on researchers' shoulders, and the included papers also discuss regulatory facilitations and instrumental support of EHR-based research (B6). The support spans from administrative instruments such as abbreviated ethics review and waivers of informed consent requirement to financial investments into technological infrastructure and political support for embedded research. Financial and infrastructure investments are necessary because EHR-based research could encounter technical difficulties with implementation with regards to amount and versatile character of data, data quality, as well as other organizational problems (B7). A separate issue that we distinguished in the included papers is factors that hinder informing participants and obtaining their informed consent. These factors are related to ineffective outreach to participants, insufficient communication with vulnerable and underprivileged groups, problems with processing and documenting consent, especially in regards to tailoring consent to individual preferences (B8).

There are also two context-dependent factors of implementation of embedded research: public awareness (B9) and researchers’ attitude (B10). The public can favor EHR-based research or oppose it. Public awareness is also associated with previous experience with EHRs and healthcare, with information policies and practices, as well as multiple individual factors such as confidence in one’s computer skills, perception of sensitivity of health data and level of risk. Also, researchers’ attitudes (B10) is also a factor that could influence the conduct of EHR-based research. Researchers recognize ethical and legal challenges of embedded research and designing research protocols and they try to balance the imperatives to benefit society and to develop science. However, sometimes they can come to a conclusion that administrative and regulatory barriers make their research projects unworthy of pursuit.

#### Data management (C11–C18)

We found several aspects of ethical data management in the literature. First of all, the included papers discuss an issue of safe and secure data storage. There is a variety of specific procedural and technical security measures (C11) such as firewalls, data safe havens and secure data access that can be deployed. Their main goal is to control access to data and protect patients’ confidentiality while at the same time streamlining legitimate research. Data used in research has different levels of identifiability (pseudonymized, de-identified, minimized data sets, aggregated data sets) at different stages of research (C12). Some elements of EHRs can contain sensitive information about a patient (C13), such as information about mental illness, fertility, face images, free text with references to third person. Also, quality, quantity and integrity of data (C14) has ethical importance, since researchers can draw conclusions that are meaningful and beneficial to wide social groups only when the data are representative and of good quality. Poor data quality can be also considered as a waste of resources. A separate problem that we discerned in the literature, is data ownership, management and curation (C15). This category encompasses an issue of data control and maintenance, and the possibility of selling data. Meaningful sharing of data (C16) means that data is a valuable resource that can be wasted, when it is not properly used, or when it is collected in such a data format that prevents sharing. We also distinguished in the literature a separate issue concerning data extraction and transfer (C17). From an ethical point of view it is important who has access to health data before it is extracted for the purpose of research, which elements of an EHRs are extracted. If the data contain sensitive information, who is responsible for storage and extraction of data: healthcare workers, researchers or any third entities, as well as who is responsible for decisions about extracting or using data of particular people or groups. This problem is linked to legitimacy of uses and users (C18). Medical professionals, researchers, private companies, healthcare providers, data institutes, disease foundations, governments and patients themselves can use EHR for various legitimate and (potentially) illegitimate purposes, such as research and development, marketing, and education. Empirical research shows (Table 2) that patients and participants sometimes insist on higher ethical scrutiny for certain users and uses. For instance, research use of EHR data by private companies can be considered less legitimate than use by university researchers.

#### Impact of digitalization on healthcare system providers’ operations and patients’ engagement (D19–D24)

Digitalization of healthcare systems and implementation of embedded research impacts healthcare providers and alters their relationship with patients. This impact of digitalization has also an ethical dimension that is discussed in the literature. First of all, EHR-based research changes professional relationships within institutions and between institutions (D19) in a way that blurs the distinction between practice, research and public health activities. Moreover, healthcare workers have to face new responsibilities and adapt to new practices in regard to data processing. As reported in the literature, digitization of health records and embedded research poses yet another challenge to communication with patients (D20), who have to be informed about data policies, including policies on data storage, sharing, and roles of a physician and provider as intermediaries between researchers. As is indicated in the literature, this process must foster trust between healthcare professionals and patients. Trust is crucial because patients should be still willing to share with their physicians all sensitive information that is important for their health. Another category that we distinguished in the included papers is directly connected with already discussed alterations in healthcare practice: digitization of health records brings new ethical responsibilities of the medical staff (D21). Healthcare professionals have to understand ethical challenges associated with EHRs and embedded research, especially if they are involved in data processing. Involvement into data processing, notably, when it directly concerns cooperation with researchers, can also be a source of moral distress and unwillingness to share patients’ data outside the context of their current care. Moreover, as reported in the analyzed papers, embedded research also requires additional work from staff and patients (D 22). Staff is involved in data curation and informing patients; patients have to respond to research invitations and fill out additional documents. In both cases, all these activities are discrete forms of resource allocation. Another aspect of embedded research, discussed in the literature, is the pivotal role of patients (D23). LHSs are sustainable in the long run only when there is broad patient participation, public acceptance and support. Some included papers addressing this issue underline the necessity of patient involvement into policy-making and implementation. Finally, a few papers included in our sample envision and discuss an idea that we called “digital patient-citizenship” (D24). Digital patient-citizenship is a proposal to encourage patient participation in research activities, research oversight, policy making and all the responsibilities associated with curation of data. In that vision, a personal EHR is a tool of patient empowerment. It is also an attempt to take a bit broader look at EHRs, not merely as an element of the healthcare system, but an element of contemporary digital culture.

#### Risk, harms and burdens of research with EHRs (E25-39)

Although usually it is believed that embedded research does not pose more than minimal risk, due to the fact that research use of EHRs is not associated with additional risk other than everyday medical practice, we found fifteen different categories of risk posed by EHR-based research. The most obvious seems to be risk to privacy (E25), then risk to patient autonomy (E26), when one loses control over one’s data. Because data use can violate a patient’s beliefs and values, it is associated with a risk of dignitary harm (E27). Risk for patients encompasses also harmful use of data (E28), e.g., when one’s data is used by a misleading pharmaceutics marketer, or when one loses a job after a leak of health information. In the papers we also encountered a risk of legal (E29) and psychological harm (E30). Some authors discuss information risk (E31) that is associated with the fact that patients could be insufficiently informed about research or that they can misunderstand provided information. Information risk category encompasses also a common situation of social science: it is difficult to study patients’ opinions and preferences, since people usually form opinions during research itself. Another category of risk that we encountered in the literature is a risk of exploitation (E32), when one’s data are used without consent for commercial purposes. In the included papers we also identify undue pressure to participate in research (E33). This kind of risk is closely associated with the risk to the whole healthcare system: when patients realize that their data are being used for illegitimate purposes or by illegitimate users that could undermine trust between the healthcare system and patients (E34) and lead to a massive drop out from research activities. Undermined trust can also result in other risks to patients. For example compromised care (E35) because patients withhold certain information from healthcare professionals; compromised care can be also a result of burdens that are associated with processing information and deflection of attention from a patient. We also distinguished a category of group mediated risk (E36). Research using EHRs provides detailed health related knowledge about individual patients, as well as about certain groups (e.g., ethnic minorities) and populations. Based on this knowledge, individuals and groups can be stigmatized and discriminated against by, for example, refusal of certain services. We also found a risk of financial conflict of interest (E37): researchers can take advantage of access to data and sell it. This kind of risk is more often mentioned by respondents in empirical research. Finally, their EHR-based research also poses risk to healthcare providers. First, because they can face additional work and administrative burden (E38). Second, disclosure of information about providers can sometimes undermine their reputation and commercial interests (E39).

#### Measures for subject protection (F40–F46)

The included papers also discuss a variety of protective measures against negative consequences of participation in EHR-based research: independent review by a research ethics committee or privacy board (F40), requirement for informed consent or authorization (F41), legal privacy regulations and ethical guidelines (F42), risk assessment procedures (F43), primary care provider consent (F44), as well as community or patients’ panel consent (F45). When one considers a healthcare institution as a participant of embedded research, then some authors also propose providers’ consent mechanism (F46) that would be devised for protection of healthcare providers’ interests, such as control of access to data.

#### Type of consent (G47–G54)

The papers that we analyzed discuss several different types of consent in the context of EHR-based research. The first problem associated with research is approaching a patient and initiation of contact (G47). Embedded research might require different levels of patient involvement and in the literature a broad variety of options spanning from no need for consent, through opt-out to fully-informed and document consent (G48) are discussed. A separate issue is documentation of consent for EHR-based research and whether it should be a written document or just a verbal consent that is then marked in a patient’s electronic documentation. The included papers discuss also a seemingly opposing approaches to consent: broad consent (G50), where a patient agrees in advance for a whole spectrum of different and unknown research belonging to a certain category, and interactive and granular consent (G51), when a patient can actively select not only studies that she wants to participate in, but also pieces of information from her EHR that she is willing to share with researchers. The papers we analyzed also discuss such issues as when should researchers be able to obtain retrospective consent (D52) or what conditions have to be met to waive this requirement. Finally, papers also mention proxy consent (G53) and assent (G54). The latter two issues, however, are not thoroughly discussed in the literature.

#### Content of consent (H55–H60)

As a separate topic we distinguished a category of content of consent (H). This category encompasses all pieces of information that a patient could or should be provided with in EHR-based research. Several of the included papers discuss issues such as the issue of data management, purpose of research, possible future use of data, storage and sharing details (H55). Some authors include additional items to the list of items that should be discussed in a consent form: security measures (H56), benefits, risks and burdens (H57) associated with research, as well as commercial application of data (H58). Only 4 papers discuss the issue of communication of study results (H59), and 2 mention that informed consent should contain information about research funding sources (H60).

#### Reasons and motives for participation in EHR-based research (I62–I64)

Empirical research on patient attitudes towards EHR-based research is summarized in Table 2. Generally, patients or respondents representing the general public express their willingness to make one’s EHR available for the sake of research, as well as express other motives, such as altruism and solidarity, support for science, and health of future generations (I61, I62). Decisions to participate in research depend on trust of involved institutions (I63) and personal and sociodemographic factors such as race, education, income, living in a city, and employment status (I64).

#### Emotions experienced as a result of reflection on EHRs and/or participation in HER-based research (J65–J67)

Mainly empirical papers allowed us to distinguish also the emotional component of attitude toward EHRs and EHR-based research. Some patients and participants express their positive emotional attitude towards research encompassing a sense of comfort, trust, and even excitement with new vistas for biomedical research (J65). Others responded with emotions indicating negative attitudes toward research, such as discomfort, wariness, lack of commitment, anxiety and confusion (J66). Finally, there were also patients and participants whose attitude was inconsistent and they expressed enthusiastic support for research and great concern for privacy, felt uncomfortable about the fact that facilitation of research decreases the level of personal control over data (J67).

#### Ethical values, rights, and obligations (K68–K74)

Finally, we identified 7 explicit ethical topics discussed in the context of EHR-based research. 49 papers discuss the traditional patient rights to information and to autonomous choice, for instance to donate one’s EHR to be used after the donor's death (K68). Next most frequently discussed issue is information, public education and public engagement (K69) that is also associated with such values as transparency, empowering of patients and communities. In the context of embedded research, the ethical principle of beneficence (K70) is also discussed. In addition, as some authors suggest, not only researchers and healthcare workers are obliged to conduct research for the public benefit, but patients are also under similar obligation to participate in low risk embedded research. Another quite frequently invoked ethical principle is the principle of justice (K71), which is translated in the context of EHR-based research into fair benefit sharing, fair recruitment and protection of vulnerable groups and individuals, and involving local communities in the process of research. A few papers discuss the problem of research integrity and respect for intellectual property (K73). Less frequently, a right to optimal healthcare and right to clinical judgment is discussed, as well as a right to compensation (K74).

## Discussion

### Ethical challenges of EHR-based research

The grid of categories cannot substitute normative discussion on how EHRs should be used in the course of research. Moreover, our grid of ethically relevant issues does not by itself inform which issues are more, and which are less, important, and how to solve a possible conflict between several ethical values. Therefore, the results of our study should be considered as a point of departure for a normative deliberation, not the conclusion or solution for problems. The main merit of our study is the presentation of how accessibility of the EHR presents itself in everyday research and health care practice, and what elements of this complicated picture bear ethical significance.

Two main questions emerge from the grid of categories. The first one considers tension between individual and societal interests in the context of population research. On the one hand, we see a trend to empower individuals in their decision making, through new digital technologies (G61). On the other hand, policy makers and researchers aim at streamlining research and relaxing obstructive regulations, seeking quick delivery of generalizable knowledge (B6). This tension between individual rights and public health interests seems to be even more poignant in the time of public health emergencies, such as COVID-19 pandemic. In our opinion, this tension can be overcome, perhaps, by the idea of digital citizenship and recognition of patient contributions to the LHS (D23–24). The idea of digital citizenship appears only in 14 papers that we analyzed. However, the concept of citizenship is a promise to reconcile autonomy of an individual citizen, whose unalienable rights should always be respected by the state, and the fact that a citizen is always a member of the political community. Citizen participation in community life aims at defining and realizing the common good. Yet, it is still a promising idea and is not clear how exactly it can be implemented into policies, regulation and practice.

The second question is: does implementation of LHSs fueled by EHRs simply exacerbate already existing ethical problems and what kind of new challenges for policymakers, healthcare providers and researchers does it create? In the following discussion, we attempt to provide some additional context for these two issues and shed some light on a possible ethical justification for selected ethical categories from the grid.

### Towards digital citizenship?

#### Granular approach to consent

Legal regulations in many countries (e.g. US and EU) already allow for modification or even a complete waiver of informed consent in the case of EHR-based research. Nevertheless, a waiver of informed consent disables individual control over data and can undermine public trust in research and healthcare institutions. The idea of empowering patients by giving them access to their data through IT tools is in line with the empirical studies that show patients want to know what is happening with their EHRs and that information policies play an important role in preserving trust towards healthcare institutions (Tale 2). Technology opens an opportunity to a more granular approach to informed consent (G51). It means that a patient, logging through a patient interface, is able to choose which elements of her records can be accessed by researchers and for how long. Such digital tools are already applied in research and clinical practice (Shelton 2011, Wallace and Miola 2021). This is in congruence with Neal Dickert’s and his colleagues analysis that distinguishes seven different functions of informed consent. Informed consent 1. Makes the process of research transparent; 2. Allows to control and authorize research; 3. Gives a patient opportunity to participate only in those research projects which conform to her values; 4. Protects and promotes welfare; 5. Promotes public trust, 6. Is required by regulations, and researchers who follow regulations are protected, and 7. Promotes research integrity (Dickert et al. 2017). Technological advances therefore give us an opportunity to balance individual control and public responsibility, because it seems reasonable to think that not all functions of informed consent have to be performed in all circumstances. There are some tradeoffs that a society or community could negotiate. For instance, one can agree that public health goals might require obligatory accessibility of health information concerning infectious diseases, especially in the time of pandemic, but other elements of an EHR could be under patient’s control.

#### Exercising control over one’s data

The empirical studies that we analyzed also reveal that some patients hold a belief that data ownership is an appropriate instrument to control their data (Table 2). The idea of health data ownership has been discussed thoroughly, especially in the US context (Evans 2011; Haislmaier 2006; Hall and Schulman 2009; Hall 2010; Kish and Topol 2015; Mirchev et al. 2020; Purtova 2015, 2017; Rodwin 2009). It was argued that every EHR as a byproduct of therapeutic encounters belongs to a patient and medical facility (Haislmaier 2006; Hall and Schulman 2009; Hall 2010). An independent intermediary—EHR bank—can manage collection, exchange and access to databases for researchers, and other parties. The revenue would then be shared among the patient, the medical facility, and the EHR bank. Thus, it was thought, an invisible hand of the market will give a spur to the economy and research enterprise at once.

Nonetheless, this idea has been criticized for several reasons. The proponents of public interests argued that privatization of EHRs would increase the cost of public health and epidemiological research, as well as result in biased, not representative, research samples (Rodwin 2009; Evans 2011). Barbara Evans argues that in the US legal context property right is not an absolute one, and in reality it does not give an individual stronger privacy protection instruments (Evans 2011).

Generally, two different regimes of data protection with some variations can be distinguished (Painsack 2019). In the first regime an individual has control over data. This control can be exercised by property rights (the US) or data can be considered, as in the EU, an inalienable individual possession protected by civil rights (Painsack 2019). The second regime of data protection introduces an element of collective control over data, and as Prainsack argues, this element of collective control can be reconciled with the concept of data as inalienable possession. A similar idea was also discussed in one article included in our sample (Grande et al. 2014). Public data stewardship is an element of digital citizenship, where the community as a whole can balance individual rights and common good through deliberation and decisions can be made in a legitimate political process. Thus the problem of who can use people’s data and how is left neither to paternalistic protection of public health nor to purely economic forces. Data stewardship, nonetheless, requires additional education efforts, and can be probably implemented in societies with a high level of public trust and solidarity (K70-71). It seems that as societies, we need digital education that would explain the benefits and risks of new technologies.

#### Healthcare provider as a research participant

The problem of data stewardship is even more complicated, because a community has to balance not only individual and societal interests, but also interests of corporate entities. Gregory Simon realizes that a healthcare provider cannot be unconditionally included into embedded research, because this kind of research also entails some risks for the whole institution (Simon et al. 2017, Piasecki & Dranseika 2021). We discussed the problem thoroughly in a separate publication, and we proposed three different strategies of finding a balance between a provider's professional obligation to contribute to the development of healthcare and duty to protect important interests of the institution. The first approach is the self-regulating model, probably the most suitable for free market driven healthcare systems, like in the US, where balancing is managed by healthcare providers themselves. The centralized model is more suitable for a centralized public healthcare system. In this model, the process of managing patients’ data is governed by a state body. The most democratic and patient- empowering approach is possible in the mediating model, where providers, the state and the citizens can negotiate what kind of data can be made accessible to researchers (Piasecki & Dranseika 2021).

### New ethical challenges for policy makers

#### Research disrupts practice

EHRs are a key element of collecting data in a systematic manner during medical practice and then using these data to develop generalizable knowledge. However, in everyday life researchers usually do not have direct access to EHRs and practitioners’ access to EHRs is strictly regulated, as well. Thus, the use of EHRs presents a set of practical and ethical challenges: who can access an actual patient’s record and to what extent? How are the data from the record extracted and stored? (Evans 2011) How does this process of data extraction for the purpose of research influence everyday healthcare provider operations? (D19–22) How to contact patients and how to provide them with information about the research projects? Moreover, the empirical research with healthcare staff that is involved in processing and sharing the data reveals that healthcare professionals are not comfortable with sharing data outside the context of healthcare (Stevenson 2015) (D21). They are trained to keep patients’ data confidential. They build their trust relationship with patients on the basis of this commitment to professional confidentiality. As it has already been mentioned, building an LHS requires, then, not only technological tweaks and contact with patients, but also changing the organizational culture of healthcare providers (Foley and Fairmichael 2015).

In a LHS healthcare professionals: physicians, nurses, administrative clerks are assigned new roles (F29, K69–72). They are becoming a part of the LHS, obliged to provide additional information to researchers, overview and assess collected data and handle contacts with researchers. These new assignments transform ethical responsibilities of healthcare workers. The healthcare professionals have to accept and internalize these new responsibilities in order to make the LHS run in an efficient and ethical way. Similarly, patients have to recognize that quality of care, effectiveness and safety of provided therapies depend on their contribution and cooperation.

#### Ethical framework for the LHS

But it seems that these relatively miniscule changes in the healthcare systems constitute a more general ethical problem. Namely, what is the role of healthcare and healthcare systems in modern societies? Is healthcare a human right and a response to individual health needs? Is healthcare just one of the different goods that are available in the free market economy? (Daniels 2001) This question is not always dealt with directly. It seems that when one discusses a new ethical framework for LHSs, this implicitly answers it (Faden et al. 2013a; b). A raw fact of altered accessibility of EHR, accumulation of data and rising computing powers change our approach to clinical ethics, research ethics and public health ethics (Piasecki & Dranseika 2019a). We are facing a stark choice: what values our regulatory and technological environment should espouse: give precedence to individual interests or promote the public good? Currently, in both clinical and research ethics, the binding principle is still that of precedence of the individual interest. The new approach seems to take a different turn and underscores the moral importance of public good. In public health ethics the main goal is good of the community as a whole. But in the pursuit of that public good we cannot entirely discard the value of individuals (Kass 2001, Piasecki & Dranseika 2019b). And this new question “What should be the organizing principle of EHRs use in LHS?” also emerges from the results of our study. This issue has its consequences on all levels of the healthcare system and it affects not only the framing regulations, but also professional roles of healthcare workers, and patients’ attitudes and expectations.

## Conclusion

In this systematic review, we have presented a wide spectrum of ethical issues involved in EHR-based research. All these problems are related to the main issue: how to manage access to health information. The reviewed literature allowed us to capture different aspects of access management and perspectives of different stakeholders. In conclusion, it can be said that most of the problems arise from a rapid cultural change. The framing concepts of privacy, as well as individual and public dimensions of beneficence, are changing. We are currently living in the middle of this transition period. Human emotions and mental habits, as well as laws, are lagging behind technological developments. In the medical tradition, individual patient’s health has always been in the center. Transformation of healthcare care, its digitalization, seems to have some impacts on our perspective on health care ethics, research ethics and public health ethics.

## Supplementary Information

Below is the link to the electronic supplementary material.

Supplementary file1 (XLSX 93 kb)

Supplementary file2 (DOCX 75 kb)

## Data Availability

All essential data are available in supplementary materials.

## Abbreviations

EHR: Electronic health records
LHS: Learning healthcare system
CCM: Constant comparative method
