# Supplementary material for: Ethical issues in biomedical research using electronic health records: a systematic review
Source: Med Health Care Philos. 2021 Jun 19;24(4):633–58. doi: 10.1007/s11019-021-10031-6 (PMC8214390; doi:10.1007/s11019-021-10031-6)
Supplement: Supplementary file 2 — Supplementary file2 (DOCX 75 kb) [file 11019_2021_10031_MOESM2_ESM.docx]

Tabela 1. Search strategy – medline-ovid

| **Number** | **Query** | **Result** |
| --- | --- | --- |
| **#1** | ethic.mp. | 2 022 |
| **#2** | exp Ethics/ | 137 097 |
| **#3** | ethics.mp. | 118 189 |
| **#4** | ethical.mp. | 71 297 |
| **#5** | bioethic.mp. | 65 |
| **#6** | bioethics.mp. | 16 976 |
| **#7** | exp Bioethics/ | 10 068 |
| **#8** | bioethical.mp. | 6 386 |
| **#9** | moral.mp. | 26 608 |
| **#10** | exp Morals/ | 157 228 |
| **#11** | morals.mp. | 13 569 |
| **#12** | morality.mp | 3 734 |
| **#13** | consent.mp. | 76 083 |
| **#14** | confidentiality.mp. | 27 112 |
| **#15** | exp Confidentiality/ | 49 324 |
| **#16** | exp Privacy/ | 13 784 |
| **#17** | privacy.mp. | 17 128 |
| **#18** | secrecy.mp. | 1 661 |
| **#19** | exp Patient Safety/ | 13 863 |
| **#20** | "patient safety".mp. | 33 273 |
| **#21** | exp Patient Rights/ | 73 198 |
| **#22** | "patient rights".mp | 7 028 |
| **#23** | exp Human Rights/ | 132 333 |
| **#24** | "human rights".mp. | 18 413 |
| **#25** | "natural rights".mp. | 23 |
| **#26** | 1 or 2 or 3 or 4 or 5 or 6 or 7 or 8 or 9 or 10 or 11 or 12 or 13 or 14 or 15 or 16 or 17 or 18 or 19 or 20 or 21 or 22 or 23 or 24 or 25 | 391236 |
| **#27** | ehr.mp. | 4 925 |
| **#28** | "electronic health record".mp. | 5 560 |
| **#29** | exp Electronic Health Records/ | 14 392 |
| **#30** | "electronic health records".mp | 17 488 |
| **#31** | "electronic medical record". mp. | 5 750 |
| **#32** | "electronic medical records".mp. | 6 607 |
| **#33** | "electronic patient record".mp. | 942 |
| **#34** | "electronic patient records".mp. | 959 |
| **#35** | "personal health record".mp. | 490 |
| **#36** | "personal health records".mp. | 529 |
| **#37** | exp Health Records, Personal/ | 1 371 |
| **#38** | "digital health record".mp. | 5 |
| **#39** | "digital health records".mp. | 10 |
| **#40** | "digital medical record".mp. | 8 |
| **#41** | "digital medical records".mp. | 26 |
| **#42** | 27 or 28 or 29 or 30 or 31 or 32 or 33 or 34 or 35 or 36 or 37 or 38 or 39 or 40 or 41 | 32 199 |
| **#43** | exp Biomedical Research/ | 241 010 |
| **#44** | "biomedical research".mp. | 72 892 |
| **#45** | "learning healthcare".mp. | 103 |
| **#46** | "learning health care".mp. | 126 |
| **#47** | exp Quality Improvement/ | 16 924 |
| **#48** | "quality improvement".mp. | 38 249 |
| **#49** | "quality improvements".mp. | 841 |
| **#50** | 42 or 43 or 44 or 45 or 46 or 47 or 48 | 286 282 |
|  | 26 and 42 and 50 | 584 |
| FINAL RESULT (22.03.2018) | | **584** |

Table 2. Search strategy - embase

| **Number** | **Query** | **Result** |
| --- | --- | --- |
| **#1** | ethic | 3 898 |
| **#2** | ethics | 212 995 |
| **#3** | 'ethics'/exp | 264 327 |
| **#4** | ethical | 92 147 |
| **#5** | #1 OR #2 OR #3 OR #4 | 333 361 |
| **#6** | bioethic | 130 |
| **#7** | bioethics | 33 114 |
| **#8** | 'bioethics'/exp | 10 643 |
| **#9** | bioethical | 2 801 |
| **#10** | #6 OR #7 OR #8 OR #9 | 34 379 |
| **#11** | moral | 26 495 |
| **#12** | morals | 750 |
| **#13** | morality | 34 023 |
| **#14** | 'morality'/exp | 32 309 |
| **#15** | #11 OR #12 OR #13 OR #14 | 48 894 |
| **#16** | consent | 141 920 |
| **#17** | confidentiality | 30 047 |
| **#18** | 'confidentiality'/exp | 25 936 |
| **#19** | privacy | 21 794 |
| **#20** | 'privacy'/exp | 13 252 |
| **#21** | secrecy | 3 887 |
| **#22** | "patient safety" | 109 711 |
| **#23** | 'patient safety'/exp | 95 252 |
| **#24** | "patient rights" | 864 |
| **#25** | "human rights" | 28 300 |
| **#26** | 'human rights'/exp | 196 721 |
| **#27** | "natural rights" | 22 |
| **#28** | #5 OR #10 OR #15 OR #16 OR #17 OR #18 OR #19 OR #20 OR #21 OR #22 OR #23 OR #24 OR #25 OR #26 OR #27 | 619 961 |
| **#29** | "electronic health record" | 11 649 |
| **#30** | 'electronic health record'/exp | 7 199 |
| **#31** | "electronic health records" | 7 666 |
| **#32** | "electronic medical record" | 45 959 |
| **#33** | 'electronic medical record'/exp | 43 042 |
| **#34** | "electronic medical records" | 12 841 |
| **#35** | "electronic patient record" | 2 538 |
| **#36** | 'electronic patient record'/exp | 989 |
| **#37** | "electronic patient records" | 2 076 |
| **#38** | "personal health record" | 572 |
| **#39** | "personal health records" | 687 |
| **#40** | "digital health record" | 11 |
| **#41** | "digital health records" | 11 |
| **#42** | "digital medical record" | 17 |
| **#43** | "digital medical records" | 58 |
| **#44** | EHR | 6 847 |
| **#45** | #29 OR #30 OR #31 OR #32 OR #33 OR #34 OR #35 OR #36 OR #37 OR #38 OR #39 OR #40 OR #41 OR #42 OR #43 OR #44 | 61 373 |
| **#46** | "biomedical research" | 128 572 |
| **#47** | "learning healthcare" | 133 |
| **#48** | "learning health care" | 207 |
| **#49** | "quality improvement" | 44 134 |
| **#50** | "quality improvements" | 1 159 |
| **#51** | #46 OR #47 OR #48 OR #49 OR #50 | 173 799 |
| **#52** | #28 AND #45 AND #51 | 429 |
| **#53** | #28 AND #45 AND #51 AND [english]/lim | 429 |
| FINAL RESULT (22.03.2018) | | **429** |

Tabela 3. search strategy – scopus

| **Number** | **Query** | **Result** |
| --- | --- | --- |
| **#1** | TITLE-ABS-KEY ( ethic ) | 277 443 |
| **#2** | TITLE-ABS-KEY ( ethics ) | 277 443 |
| **#3** | TITLE-ABS-KEY ( ethical ) | 153 493 |
| **#4** | TITLE-ABS-KEY ( bioethic ) | 156 |
| **#5** | TITLE-ABS-KEY ( bioethics ) | 24 128 |
| **#6** | TITLE-ABS-KEY ( bioethical ) | 7 808 |
| **#7** | TITLE-ABS-KEY ( moral ) | 116 281 |
| **#8** | TITLE-ABS-KEY ( morals ) | 116 281 |
| **#9** | TITLE-ABS-KEY ( morality ) | 50 382 |
| **#10** | TITLE-ABS-KEY ( consent ) | 117 691 |
| **#11** | TITLE-ABS-KEY ( confidentiality ) | 48 893 |
| **#12** | TITLE-ABS-KEY ( privacy ) | 96 174 |
| **#13** | TITLE-ABS-KEY ( secrecy ) | 15 139 |
| **#14** | TITLE-ABS-KEY ( "patient safety" ) | 103 083 |
| **#15** | TITLE-ABS-KEY ( "patient rights" ) | 18 439 |
| **#16** | TITLE-ABS-KEY ( "human rights" ) | 87 064 |
| **#17** | TITLE-ABS-KEY ( "natural rights" ) | 877 |
| **#18** | ( TITLE-ABS-KEY ( ethic ) )  OR  ( TITLE-ABS-KEY ( ethics ) )  OR  ( TITLE-ABS-KEY ( ethical ) ) | 348 781 |
| **#19** | ( TITLE-ABS-KEY ( bioethic ) )  OR  ( TITLE-ABS-KEY ( bioethics ) )  OR  ( TITLE-ABS-KEY ( bioethical ) )  OR  ( TITLE-ABS-KEY ( moral ) )  OR  ( TITLE-ABS-KEY ( morals ) )  OR  ( TITLE-ABS-KEY ( morality ) ) | 157 131 |
| **#20** | ( TITLE-ABS-KEY ( consent ) )  OR  ( TITLE-ABS-KEY ( confidentiality ) )  OR  ( TITLE-ABS-KEY ( privacy ) )  OR  ( TITLE-ABS-KEY ( secrecy ) ) | 253 144 |
| **#21** | ( TITLE-ABS-KEY ( "patient safety" ) )  OR  ( TITLE-ABS-KEY ( "patient rights" ) )  OR  ( TITLE-ABS-KEY ( "human rights" ) )  OR  ( TITLE-ABS-KEY ( "natural rights" ) ) | 205 945 |
| **#22** | ( ( TITLE-ABS-KEY ( ethic ) )  OR  ( TITLE-ABS-KEY ( ethics ) )  OR  ( TITLE-ABS-KEY ( ethical ) ) )  OR  ( ( TITLE-ABS-KEY ( bioethic ) )  OR  ( TITLE-ABS-KEY ( bioethics ) )  OR  ( TITLE-ABS-KEY ( bioethical ) )  OR  ( TITLE-ABS-KEY ( moral ) )  OR  ( TITLE-ABS-KEY ( morals ) )  OR  ( TITLE-ABS-KEY ( morality ) ) )  OR  ( ( TITLE-ABS-KEY ( consent ) )  OR  ( TITLE-ABS-KEY ( confidentiality ) )  OR  ( TITLE-ABS-KEY ( privacy ) )  OR  ( TITLE-ABS-KEY ( secrecy ) ) )  OR  ( ( TITLE-ABS-KEY ( "patient safety" ) )  OR  ( TITLE-ABS-KEY ( "patient rights" ) )  OR  ( TITLE-ABS-KEY ( "human rights" ) )  OR  ( TITLE-ABS-KEY ( "natural rights" ) ) ) | 822 033 |
| **#23** | TITLE-ABS-KEY ( ehr ) | 7 893 |
| **#24** | TITLE-ABS-KEY ( "electronic health record" ) | 23 595 |
| **#25** | TITLE-ABS-KEY ( "electronic health records" ) | 23 595 |
| **#26** | TITLE-ABS-KEY ( "electronic medical record" ) | 35 256 |
| **#27** | TITLE-ABS-KEY ( "electronic medical records" ) | 35 256 |
| **#28** | TITLE-ABS-KEY ( "electronic patient record" ) | 3 045 |
| **#29** | TITLE-ABS-KEY ( "electronic patient records" ) | 3 045 |
| **#30** | TITLE-ABS-KEY ( "personal health record" ) | 1 819 |
| **#31** | TITLE-ABS-KEY ( "personal health records" ) | 1 819 |
| **#32** | TITLE-ABS-KEY ( "digital health records" ) | 34 |
| **#33** | TITLE-ABS-KEY ( "digital health record" ) | 34 |
| **#34** | TITLE-ABS-KEY ( "digital medical record" ) | 56 |
| **#35** | TITLE-ABS-KEY ( "digital medical records" ) | 56 |
| **#36** | ( TITLE-ABS-KEY ( ehr ) )  OR  ( TITLE-ABS-KEY ( "electronic health record" ) )  OR  ( TITLE-ABS-KEY ( "electronic health records" ) )  OR  ( TITLE-ABS-KEY ( "electronic medical record" ) )  OR  ( TITLE-ABS-KEY ( "electronic medical records" ) )  OR  ( TITLE-ABS-KEY ( "electronic patient record" ) )  OR  ( TITLE-ABS-KEY ( "electronic patient records" ) ) | 50 678 |
| **#37** | ( TITLE-ABS-KEY ( "personal health record" ) )  OR  ( TITLE-ABS-KEY ( "personal health records" ) )  OR  ( TITLE-ABS-KEY ( "digital health records" ) )  OR  ( TITLE-ABS-KEY ( "digital health record" ) )  OR  ( TITLE-ABS-KEY ( "digital medical record" ) )  OR  ( TITLE-ABS-KEY ( "digital medical records" ) ) | 1 902 |
| **#38** | ( ( TITLE-ABS-KEY ( ehr ) )  OR  ( TITLE-ABS-KEY ( "electronic health record" ) )  OR  ( TITLE-ABS-KEY ( "electronic health records" ) )  OR  ( TITLE-ABS-KEY ( "electronic medical record" ) )  OR  ( TITLE-ABS-KEY ( "electronic medical records" ) )  OR  ( TITLE-ABS-KEY ( "electronic patient record" ) )  OR  ( TITLE-ABS-KEY ( "electronic patient records" ) ) )  OR  ( ( TITLE-ABS-KEY ( "personal health record" ) )  OR  ( TITLE-ABS-KEY ( "personal health records" ) )  OR  ( TITLE-ABS-KEY ( "digital health records" ) )  OR  ( TITLE-ABS-KEY ( "digital health record" ) )  OR  ( TITLE-ABS-KEY ( "digital medical record" ) )  OR  ( TITLE-ABS-KEY ( "digital medical records" ) ) ) | 51 717 |
| **#39** | TITLE-ABS-KEY ( "biomedical research" ) | 63 498 |
| **#40** | TITLE-ABS-KEY ( "learning healthcare" ) | 120 |
| **#41** | TITLE-ABS-KEY ( "learning health care" ) | 135 |
| **#42** | TITLE-ABS-KEY ( "quality improvement" ) | 59 773 |
| **#43** | TITLE-ABS-KEY ( "quality improvements" ) | 59 773 |
| **#44** | ( TITLE-ABS-KEY ( "biomedical research" ) )  OR  ( TITLE-ABS-KEY ( "learning healthcare" ) )  OR  ( TITLE-ABS-KEY ( "learning health care" ) )  OR  ( TITLE-ABS-KEY ( "quality improvement" ) )  OR  ( TITLE-ABS-KEY ( "quality improvements" ) ) | 123 146 |
| **#45** | ( TITLE-ABS-KEY ( ethic )  OR  TITLE-ABS-KEY ( ethics )  OR  TITLE-ABS-KEY ( ethical )  OR  TITLE-ABS-KEY ( bioethic )  OR  TITLE-ABS-KEY ( bioethics )  OR  TITLE-ABS-KEY ( bioethical )  OR  TITLE-ABS-KEY ( moral )  OR  TITLE-ABS-KEY ( morals )  OR  TITLE-ABS-KEY ( morality )  OR  TITLE-ABS-KEY ( consent )  OR  TITLE-ABS-KEY ( confidentiality )  OR  TITLE-ABS-KEY ( privacy )  OR  TITLE-ABS-KEY ( secrecy )  OR  TITLE-ABS-KEY ( "patient safety" )  OR  TITLE-ABS-KEY ( "patient rights" )  OR  TITLE-ABS-KEY ( "human rights" )  OR  TITLE-ABS-KEY ( "natural rights" ) )  AND  ( ( ( TITLE-ABS-KEY ( ehr ) )  OR  ( TITLE-ABS-KEY ( "electronic health record" ) )  OR  ( TITLE-ABS-KEY ( "electronic health records" ) )  OR  ( TITLE-ABS-KEY ( "electronic medical record" ) )  OR  ( TITLE-ABS-KEY ( "electronic medical records" ) )  OR  ( TITLE-ABS-KEY ( "electronic patient record" ) )  OR  ( TITLE-ABS-KEY ( "electronic patient records" ) ) )  OR  ( ( TITLE-ABS-KEY ( "personal health record" ) )  OR  ( TITLE-ABS-KEY ( "personal health records" ) )  OR  ( TITLE-ABS-KEY ( "digital health records" ) )  OR  ( TITLE-ABS-KEY ( "digital health record" ) )  OR  ( TITLE-ABS-KEY ( "digital medical record" ) )  OR  ( TITLE-ABS-KEY ( "digital medical records" ) ) ) )  AND  ( ( TITLE-ABS-KEY ( "biomedical research" ) )  OR  ( TITLE-ABS-KEY ( "learning healthcare" ) )  OR  ( TITLE-ABS-KEY ( "learning health care" ) )  OR  ( TITLE-ABS-KEY ( "quality improvement" ) )  OR  ( TITLE-ABS-KEY ( "quality improvements" ) ) ) | 543 |
| **#46** | ( TITLE-ABS-KEY ( ethic )  OR  TITLE-ABS-KEY ( ethics )  OR  TITLE-ABS-KEY ( ethical )  OR  TITLE-ABS-KEY ( bioethic )  OR  TITLE-ABS-KEY ( bioethics )  OR  TITLE-ABS-KEY ( bioethical )  OR  TITLE-ABS-KEY ( moral )  OR  TITLE-ABS-KEY ( morals )  OR  TITLE-ABS-KEY ( morality )  OR  TITLE-ABS-KEY ( consent )  OR  TITLE-ABS-KEY ( confidentiality )  OR  TITLE-ABS-KEY ( privacy )  OR  TITLE-ABS-KEY ( secrecy )  OR  TITLE-ABS-KEY ( "patient safety" )  OR  TITLE-ABS-KEY ( "patient rights" )  OR  TITLE-ABS-KEY ( "human rights" )  OR  TITLE-ABS-KEY ( "natural rights" ) )  AND  ( ( ( TITLE-ABS-KEY ( ehr ) )  OR  ( TITLE-ABS-KEY ( "electronic health record" ) )  OR  ( TITLE-ABS-KEY ( "electronic health records" ) )  OR  ( TITLE-ABS-KEY ( "electronic medical record" ) )  OR  ( TITLE-ABS-KEY ( "electronic medical records" ) )  OR  ( TITLE-ABS-KEY ( "electronic patient record" ) )  OR  ( TITLE-ABS-KEY ( "electronic patient records" ) ) )  OR  ( ( TITLE-ABS-KEY ( "personal health record" ) )  OR  ( TITLE-ABS-KEY ( "personal health records" ) )  OR  ( TITLE-ABS-KEY ( "digital health records" ) )  OR  ( TITLE-ABS-KEY ( "digital health record" ) )  OR  ( TITLE-ABS-KEY ( "digital medical record" ) )  OR  ( TITLE-ABS-KEY ( "digital medical records" ) ) ) )  AND  ( ( TITLE-ABS-KEY ( "biomedical research" ) )  OR  ( TITLE-ABS-KEY ( "learning healthcare" ) )  OR  ( TITLE-ABS-KEY ( "learning health care" ) )  OR  ( TITLE-ABS-KEY ( "quality improvement" ) )  OR  ( TITLE-ABS-KEY ( "quality improvements" ) ) )  AND  ( LIMIT-TO ( LANGUAGE ,  "English" ) ) | 538 |
| FINAL RESULT (22.03.2018) | | **538** |
